# Exploring Use Patterns and Racial and Ethnic Differences in Real Time Affective States During Social Media Use Among a Clinical Sample of Adolescents With Depression: Prospective Cohort Study

**DOI:** 10.2196/30900

**Published:** 2022-05-12

**Authors:** Cameron Nereim, David Bickham, Michael Rich

**Affiliations:** 1 Division of Adolescent Medicine University of South Florida Morsani College of Medicine Tampa, FL United States; 2 Division of Adolescent & Young Adult Medicine Boston Children's Hospital Harvard Medical School Boston, MA United States

**Keywords:** depression, race/ethnicity, ecological momentary assessment, internet, mental health, mood, social media, mobile phone

## Abstract

**Background:**

Increasing youth mental health problems over time correlate with increasing rates of social media use (SMU); however, a proposed contributory relationship remains unproven. To better understand how SMU impacts mental health requires a more nuanced understanding of the relationship between different patterns of SMU and specific individual factors. Studies suggest that more *active* forms of SMU may offer mental health benefits when compared with more *passive* forms. Furthermore, the literature suggests important differences in patterns of SMU and affective states among those identifying as racial and ethnic minorities.

**Objective:**

Using ecological momentary assessment (EMA), this study aims to investigate potential differences in affective states during *active* and *passive* forms of SMU and whether such differences vary by race and ethnicity.

**Methods:**

We recruited patients seeking care at a large urban adolescent medicine clinic who exhibited at least mild depressive symptoms based on Patient Health Questionnaire-9 (PHQ-9) scores. Participants completed an enrollment survey and a 7-day EMA protocol, receiving 5 EMA questionnaires per day, which assessed real time SMU behaviors and affective states using the Positive and Negative Affect Schedule–Expanded form subscales. To correct for nonindependent data with EMA responses clustered within individuals, data were analyzed using mixed-effects modeling, allowing for a random intercept at the individual level to examine associations between EMA-reported SMU and affective states while adjusting results for age, gender, race and ethnicity, PHQ-9 score, and EMA response rate.

**Results:**

A racially and ethnically diverse group of 55 adolescents aged 14 to 19 years provided a total of 976 EMA responses, averaging 17.76 (SD 8.76) responses per participant, with a response rate of 51.15%. Participants reported higher mean levels of negative affect during *active* SMU (*F*_1,215_=3.86; SE 0.05; *t*_1,215_=1.96; *P*=.05) and lower mean levels of positive affect during *passive* SMU (*F*_1,369_=3.90; SE 0.09; *t*_1,369_=–1.98; *P*=.049). However, within different racial and ethnic groups, higher levels of negative affect during moments of *active* SMU were seen only among Black non-Hispanic participants: *F*_1,81_=6.31; SE 0.05; *t*_81_=2.51; *P*=.01). Similarly, lower levels of positive affect during *passive* SMU were seen only among White non-Hispanic participants (*F*_1,295_=10.52; SE 0.13; *t*_295_=–3.24; *P*=.001).

**Conclusions:**

Although in aggregate, adolescents with depressive symptoms experienced more negative affect during *active* SMU and less positive affect during *passive* SMU, these mean outcomes were driven solely by greater negative affect during *active* SMU by Black non-Hispanic participants and lower positive affect during *passive* SMU by White non-Hispanic participants. Differences in intentionality, content, context, and expectations of SMU among youths across racial and ethnic groups may result in different affective outcomes. Exploration of the interactions among cultural differences in SMU strategies and characteristics will be critical to furthering our understanding of the impact of SMU on youth mental health.

## Introduction

### Background

Social media use (SMU), the use of “internet-based networks that enable users to interact with others, verbally and visually,” [1] is exceedingly popular among youths as it combines their desire to connect with peers with their interest in engaging technologies. Increasing rates of SMU over time [2-4] have been correlated with rising rates of youth mental health problems [5-7]; however, a proposed contributory relationship remains unproven. Much attention has been paid to the potential link between SMU and rates of major depression [8], with the latter having increased dramatically in the United States and abroad over the past one to two decades, particularly among adolescents and young women [9,10].

Although some evidence suggests that children and adolescents with high-frequency SMU experience increased rates of mental health problems and greater depressive symptoms, the overall data are inconclusive, with some studies demonstrating no difference or even suggesting minor psychological benefits [11,12]. A recent study examined 6 systematic reviews from the past decade, 3 large-scale national cohort studies, and 5 diary and ecological momentary assessment (EMA) studies examining associations between SMU and adolescent mental health [13]. Although some studies demonstrated negative associations with small effect sizes, the larger picture revealed a combination of mixed, null, and positive associations between SMU and mental health. One of the reviews demonstrated that SMU accounted for only 0.4% of the variation in well-being, paling in comparison with factors such as family socioeconomic status, family history of depression or anxiety, and exposure to adverse childhood experiences, which account for 5% to 20% of the differences in mental health symptoms [14,15]. Another large-scale review examined >80 key studies, systematic reviews, and meta-analyses, concluding that there exists a small, negative association between SMU and psychological well-being but emphasizing the ongoing need for higher-quality data with a more nuanced view of different types of SMU and the role of potential confounders [12].

Variations in the relationships between SMU and mental health outcomes may have resulted from how SMU was defined and measured, what mental health symptoms were considered, and the potential influence of confounders. When SMU is considered as a binary or duration-of-use variable, without discriminating among user characteristics or their specific SMU behaviors, a more granular and useful understanding of how SMU can affect mental health may be lost.

Most SMU studies thus far have relied on retrospective self-reports, which may experience recall bias. Studies that incorporate EMA, which allows for *in-the-moment* data collection by asking participants to report on recent lived experiences, can produce more reliable and complete data, capturing specific patterns of SMU [16].

### Patterns of SMU Characteristics

Some recent studies have taken a more nuanced look at different patterns of SMU and their relationship with specific mental health outcomes [17,18]. Evidence suggests that certain SMU characteristics may help determine whether one experiences any SMU-related negative mental health consequences. For instance, individuals who report using greater numbers of social media platforms or longer durations of SMU are more likely to experience depression or other mental health problems [19-21]. Similarly, individuals whose SMU comprises more negative interactions or who perceive themselves to be less popular or successful than their peers on social media experience decreased levels of happiness [22-25].

Regarding individual patterns of use, SMU that is *active* rather than *passive* has been associated with improved mental health outcomes [26-29]. *Active* SMU, which is characterized by the act of engaging in direct interactions with other users, sharing life experiences, or creating new content, is associated with decreased depressive symptoms in adults. As a potential explanation, *active* SMU is theorized to lead to an improved sense of well-being by increasing one’s social capital among acquaintances and eliciting more emotional support and positive feedback from friends [30]. An alternative explanation is that individuals who are less depressed may be more likely to engage in *active* SMU.

On the other hand, *passive* SMU, characterized by the act of *lurking* or observing while maintaining low engagement with other users, is associated with higher levels of depressive symptoms [28,29,31,32]. *Passive* SMU may result in more instances of upward social comparison, in which one negatively evaluates oneself in comparison with the perceived realities of others [33]. Alternatively, individuals who are more depressed may be more likely to engage in *passive* SMU. Therefore, when studying the relationship between SMU and mental health outcomes, distinguishing between *active* versus *passive* SMU is likely an important consideration.

### Individual Characteristics

#### Overview

At present, it remains unclear how the characteristics of individuals using social media may affect their frequency or type of SMU or lead to different mental health outcomes. Although there are reasons to hypothesize a contributory link between certain types of SMU and declining mental health (eg, SMU that displaces face-to-face social interactions and leads to increased social isolation and loneliness), underlying mental or behavioral health issues may actually drive high-frequency SMU. In fact, young people with certain behavioral health conditions—especially attention-deficit/hyperactivity disorder, social anxiety disorder, autism spectrum disorder, and depression—use screen media more and are predisposed to *problematic interactive media use*, characterized by a clinically relevant decline in one’s functional status [34-42]. Underlying mental health conditions may also amplify or otherwise alter the effects of SMU. Therefore, youths with depression may be at an increased risk of experiencing mental health problems related to SMU, and research focusing on this group could reveal important susceptibilities.

#### Differences in SMU by Race and Ethnicity

Multiple studies have suggested that race/ethnicity may be an important moderator of the impact of SMU on mental health. Different experiences using social media networks may confer different levels of risk by race/ethnicity for mental health outcomes. Although adolescents of different racial/ethnic groups do not differ in their total number of *social media ties* (ie, *friends* on social media platforms), Black youths report a higher number of *weak ties* (ie, low perceived level of closeness) and White youths report a higher number of *strong ties* (ie, high perceived level of closeness). Furthermore, when considering past patterns of SMU among college students, Black and Latinx students reported more web-based social media content creation than White students, despite having historically less access to the internet [43,44]. Increased content creation, as an *active* form of SMU, may contribute to some mental health benefits. Therefore, social media platforms may provide different levels of social support and mental health protection for these groups [45].

Conversely, minority groups face potential exposure to racist and/or discriminatory content during SMU. Among Black young adults, SMU on certain platforms is associated with increased anticipatory race-related stress, bodily alarm responses, and anger expression, in part mediated by individual experiences of perceived racism and everyday discrimination [46]. Higher levels of discrimination during SMU are associated with increased symptoms of depression and anxiety among young Latino adults, although not among young Latina adults [47]. SMU may expose Black individuals and other racial/ethnic minority groups to discriminatory content not experienced by their White peers, resulting in poorer mental health outcomes [48,49].

### Goals of This Study

This study has 3 aims. The first is to investigate, within a sample of youths with depression, momentary associations between SMU and three types of affective states: positive affect, negative affect, and sadness. The second aim is to investigate whether adolescents’ *active* and *passive* SMU are related to these affective states. The third aim is to determine whether there are racial/ethnic differences in momentary affective states during SMU and in the associations between SMU and affect.

## Methods

### Participants, Design, and Study Procedures

#### Overview

This study used EMA survey data from a clinical sample of patients aged 14 to 19 years recruited from a large urban adolescent medicine clinic during the period from August 2016 to March 2018. Patients seeking well care were asked to complete the Patient Health Questionnaire-9 (PHQ-9), and those with a score of ≥5, indicating at least *mild depression* as per PHQ-9 scoring guidelines [50], were eligible for inclusion. The data for this study were collected to answer a series of questions investigating youth media use behaviors and individual user characteristics, with this study answering specific questions about the role of racial/ethnic differences in use behaviors and affective experiences during SMU.

#### Ethics Approval

The institutional review board of Boston Children’s Hospital approved this study (Institutional Review Board protocol number IRB-P00019244), and participants were assured of their confidentiality. All participants or parents of minor participants provided written informed consent. Once enrolled, participants completed surveys at the time of enrollment and EMA questionnaires multiple times a day for the next 7 days.

#### Enrollment Survey

After completing the PHQ-9 as a screener for inclusion, qualifying participants completed an enrollment survey that assessed key demographic information. Consistent with the most recent US national census questionnaire, race/ethnicity/ancestry (hereafter referred to as *race/ethnicity*) was assessed with a single item that asked, “Which of the following best describes you?” with the ability to select all answers that apply from the following options: (1) *White/Caucasian*; (2) *Hispanic, Latino/a, or Spanish*; (3) *Black*; (4) *African American*; (5) *Asian or Asian American*; (6) *Middle-Eastern or North African*; (7) *Native American/American Indian/Alaskan Native*; (8) *Native Hawaiian/Other Pacific Islander*; or (9) *other, please specify*. For the purpose of our analyses, participants were grouped by self-identified race/ancestry (eg, White, Black or African American, biracial) and ethnic background (ie, Hispanic or non-Hispanic origin). The 3 primary groups identified were Black non-Hispanic, White non-Hispanic, and Hispanic.

#### EMA Assessment

##### Overview

For the week following enrollment and based on standard EMA protocol, participants received 5 daily EMA assessments administered at random intervals sent to their personal smartphones (or 1 provided to them) using MetricWire [51] software package. Notifications for surveys were sent from 8 AM to 10 PM on weekends, from 6 AM to 8 AM, and then again from 3 PM to 10 PM on weekdays. Each EMA survey assessed whether participants engaged in a series of social behaviors, including SMU, texting/asynchronous messaging, making voice/video calls, or having face-to-face conversations and concurrent affective states.

##### Capturing Real Time SMU

SMU behaviors reported in EMA surveys were characterized with respect to frequency and type of use. For the purpose of this study, SMU was defined as any behavior for which the participant indicated using a web-based platform that allows for interaction and communication. This included behaviors such as *checking a social network*, *checking a web-based bulletin board or asynchronous messaging* (eg, text, email, Snapchat, or WhatsApp), and *real time messaging* (eg, web-based chats, Skype, FaceTime, telephone calls, and messaging or chatting while playing a video game). Whenever a participant reported an instance of SMU, they were asked to answer follow-up questions about whether they were interacting or messaging with anyone to further characterize their behaviors. *Active* SMU was defined as any moment of reported SMU that included direct engagement with another person or persons via posting, responding to a post, messaging, or chatting. Conversely, *passive* SMU was defined as use that did not include direct engagement with another person (eg, reading posts or scrolling without commenting). A final term, *any* SMU, was used to describe any moment that comprised either *active* or *passive* SMU.

##### Capturing Real Time Affective States With the Positive and Negative Affect Schedule–Expanded Form Subscales

Each EMA survey assessed emotional affect using subscales of the Positive and Negative Affect Schedule–Expanded form, which measured participants’ positive affect, negative affect, and sadness [52,53]. As a tool, the Positive and Negative Affect Schedule–Expanded form is widely used and well-validated for measuring an individual’s emotional states that fluctuate over time. The subscales for positive affect (eg, attentive, alert, excited, and enthusiastic) and negative affect (eg, hostile, irritable, ashamed, and distressed) each comprise 10 items with potential responses ranging from 0 to 4 (0=very slightly or not at all and 4=extremely). The sadness subscale comprises 5 items, with potential responses ranging from 0 to 4. These subscales vary independently, meaning that negative affect does not necessarily increase when positive affect decreases and vice versa.

### Statistical Modeling and Analysis

#### Real Time Affective States During SMU

We used the EMA responses to compare reported levels of positive affect, negative affect, and sadness during SMU moments and non-SMU moments. To correct for nonindependence of the data with EMA responses clustered within an individual, we used mixed-effects modeling, allowing for a random intercept at the individual level to examine associations between SMU and EMA-reported affective scores while adjusting the results for age, gender, race/ethnicity, PHQ-9 score, and EMA response rate. We used similar procedures to examine differences in affect during *active* and *passive* SMU.

#### Real Time Affective States During SMU by Race/Ethnicity

To investigate the differences in the associations between SMU and affect by race/ethnicity, the abovementioned mixed-effects models *active*, *passive*, or *any* SMU were analyzed separately with the inclusion of an interaction term, *race/ethnicity×SMU behavior*. As this was an interaction between a 2-category (race/ethnicity) and 3-category variable (*active* or *passive* SMU vs any other behavior), it was represented in the model by 2 interaction terms. When either of these 2 variables was significant at *P*<.10, pairwise contrasts based on estimated means adjusted for the covariates were compared to determine the direction of the interaction.

## Results

### Sample Characteristics

The sample was mostly female and comprised similar percentages of Black non-Hispanic, White non-Hispanic, and Hispanic participants (Table 1). Approximately 5% (3/55) of participants were excluded from the analysis because of a lack of EMA data. Overall, participants responded to more than half of the EMA prompts. The average PHQ-9 score from enrollment revealed moderate amounts of depressive symptoms with a normal distribution. On the basis of the EMA survey data, participants reported SMU during 14.2% (139/976) of EMA responses, texting/asynchronous messaging during 13.8% (135/975) of EMA responses, making a voice/video call during 2.4% (23/975) of EMA responses, and having a face-to-face conversation during 19% (185/975) of EMA responses.

**Table 1:** Sample characteristics.

Characteristics					Values
**Participants (N=55)**					
	Age (years), mean (SD; range)			17.42 (1.50; 14-19)	
	Gender (female), n (%)			37 (67)	
	**Ethnicity, n (%)**				
		Black non-Hispanic	16 (29)		
		White non-Hispanic	15 (27)		
		Hispanic	15 (27)		
		Mixed race or other	8 (15)		
	PHQ-9^a^ score, mean (SD)			11.27 (5.26)	
**EMA^b^ responses (N=976)**					
	**EMA on SMU^c^ (total), n (%)**				
		Active SMU	95 (9.7)		
		Passive SMU	41 (4.2)		
	Response rate (%)			51.15	
	EMA responses per participant, mean (SD)			17.76 (8.76)	

^a^PHQ-9: Patient Health Questionnaire-9.

^b^EMA: ecological momentary assessment.

^c^SMU: social media use.

### EMA Outcomes

#### Affect During SMU

When comparing moments with *active*, *passive*, or *any* type of SMU with non-SMU moments using generalized linear modeling, there were some observed differences in overall real time affective states (Table 2). During moments of *any* SMU, participants reported higher overall levels of both negative affect (*F_1,131_*=5.30; SE 0.04; t_131_=2.30 [2-tailed]; *P*=.02) and sadness (*F_1,39_*=4.59; SE 0.08; t_39_=2.14; *P*=.04). During moments of *active* SMU, participants reported higher overall levels of negative affect (*F_1,215_*=3.86; SE 0.05; t_215_=1.96; *P*=.05) and trended toward higher levels of sadness. However, during moments of *passive* SMU, participants instead reported lower levels of positive affect (*F_1,369_*=3.90; SE 0.09; t_369_=–1.98; *P*=.049) without apparent differences in negative affect or sadness.

**Table 2:** Effects of SMU^a^ type on mean affect scores^b^.

SMU type and affect type			Coefficient		SE		*t* test (*df*)^c^		Significance, *P* value		95% CI
**Any**											
	Negative affect	0.08		0.04		2.30 (131)		.02		0.01 to 0.16	
	Positive affect	–0.10		0.10		–0.94 (55)		.35		–0.30 to 0.11	
	Sadness	0.16		0.08		2.14 (39)		.04		0.01 to 0.32	
**Active**											
	Negative affect	0.09		0.05		1.96 (215)		.05		0.00 to 0.18	
	Positive affect	–0.04		0.13		–0.30 (40)		.77		–0.31 to 0.23	
	Sadness	0.17		0.10		1.74 (154)		.08		–0.02 to 0.04	
**Passive**											
	Negative affect	0.03		0.06		0.46 (256)		.64		–0.09 to 0.14	
	Positive affect	–0.18		0.09		–1.98 (369)		.049		–0.35 to 0.001	
	Sadness	0.04		0.11		0.39 (17)		.70		–0.20 to 0.28	

^a^SMU: social media use.

^b^The above results are from mixed-effects modeling with adjustments for age, sex, race/ethnicity, Patient Health Questionnaire-9 score, and ecological momentary assessment response rate.

^c^2-tailed.

#### Affect During SMU by Race/Ethnicity

Using generalized linear modeling with the addition of a race×SMU type interaction term, there were some observed differences in reported affective states by race/ethnicity during moments of *active* and *passive* SMU compared with all other moments. During the moments of *active* SMU, the race×*active* SMU interaction term approached significance (Table 3). Among different racial/ethnic groups, Black non-Hispanic participants reported higher levels of negative affect during moments of *active* SMU (*F*_1,81_=6.31; SE 0.05; t_81_=2.51; *P*=.01), which was not observed for participants from other racial/ethnic groups. Notably, a similar trend in the direction of change that did not rise to the level of significance was seen for Hispanic participants, whereas no such trend was observed for White non-Hispanic participants.

During the moments of *passive* SMU, the race×*passive* SMU interaction term was also found to be significant (Table 3). Within different racial/ethnic groups, White non-Hispanic participants reported lower levels of positive affect during moments of *passive* SMU (*F*_1,295_=10.52; SE 0.13 t_295_=–3.24; *P*=.001), a finding not seen in Black non-Hispanic or Hispanic participants.

**Table 3:** Group differences in mean affect during SMU^a^ versus all other moments^b^.

Affect and race/ethnicity^a^			Coefficient		Mean difference		SE		*t* test (*df*)		Significance, *P* value		95% CI
**Negative affect (during active SMU)**													
	Race and ethnicity×active SMU (1)	0.125		N/A^c^		0.07		1.89 (132)		.06		–0.01 to 0.26	
	Race/ethnicity×active SMU (2)	0.012		N/A		0.11		0.12 (132)		.91		–0.20 to 0.22	
	Black non-Hispanic	N/A		0.13		0.05		2.51 (81)		.01		0.03 to 0.24	
	White non-Hispanic	N/A		0.01		0.04		0.18 (118)		.86		–0.08 to 0.09	
	Hispanic	N/A		0.12		0.09		1.33 (144)		.19		–0.06 to 0.30	
**Positive affect (during passive SMU)**													
	Race/ethnicity×passive SMU (1)	0.405		N/A		0.18		2.24 (677)		.03		0.05 to 0.76	
	Race/ethnicity×passive SMU (2)	0.047		N/A		0.24		0.20 (677)		.84		–0.42 to 0.52	
	Black non-Hispanic	N/A		–0.03		0.12		–0.22 (586)		.82		–0.26 to 0.21	
	White non-Hispanic	N/A		–0.43		0.13		–3.24 (295)		.001		–0.69 to –0.17	
	Hispanic	N/A		–0.07		0.20		–0.36 (868)		.72		–0.48 to 0.33	

^a^SMU: social media use.

^b^The abovementioned results, including the mean differences, are adjusted for age, gender, race/ethnicity, Patient Health Questionnaire-9 score, and ecological momentary assessment response rate. Mean differences represent average affect during SMU type (*active* or *passive*) minus average affect during any other activity.

^c^N/A: not applicable.

## Discussion

### Principal Findings

In summary, during moments of *active* SMU, participants reported higher overall levels of negative affect and sadness. However, this relationship was observed only among Black non-Hispanic participants and was not observed in White non-Hispanic participants. Similarly, during moments of *passive* SMU, participants reported lower overall levels of positive affect, and this finding was observed only among White non-Hispanic participants and not among Black non-Hispanic or Hispanic participants.

### Considering Different SMU Experiences by Race/Ethnicity

This study attempts to enrich our understanding of the complex interrelations between SMU and affective states. Previous research has revealed that the association between SMU and indicators of mental health varies considerably among individual youths [54]; however, our understanding of what drives these differences is extremely limited. Our findings indicate that race may be an individual characteristic that determines the associations between SMU and mental health. Using our full sample, we found that certain types of SMU were linked to more sadness and negative affect and less positive affect. Through follow-up interaction analyses, we discovered that these results hold true only for specific racial/ethnic groups in our sample.

Differences in media and SMU by ethnicities were well-documented during the emergence of mobile and social media, as minority youths (ie, Black, Hispanic, and Asian) were shown to consume 4.5 hours a day more media than their White peers and were more likely to be early users of social networking sites [55]. The size, closeness, and racial homogeneity of web-based social networks may differ across racial and ethnic groups. Although Black and White youths were shown to have similar numbers of friends on Facebook (when it was the dominant social media platform for youths), Black youths had weaker ties among those relationships, indicating an effort to diversify their social network and potentially increase their social capital [45]. Social media offers ethnic minorities a dualistic environment: the opportunity to develop a supportive community of similar peers tempered by increased levels of cyberaggression driven by racism [56]. These opposing forces shape the web-based communication of Black youths into experiences that likely vary greatly from those of White youths. Although our findings provide evidence that racial/ethnic differences are apparent in SMU and possible mental health effects, this area remains understudied. Additional research may help reveal how web-based experiences translate into different affectual and mental health experiences across racial/ethnic groups.

Our findings regarding differences in affect during *active* and *passive* SMU may be most informative in enhancing our understanding of different ethnic/racial groups’ experiences in web-based interactions. For White youths in our sample, *passive* SMU was linked to lower levels of concurrent positive affect. This result is consistent with other research showing that lurking, scrolling, and other passive uses of social media are linked to more symptoms of depression [29]. It is notable that lower levels of positive affect more aptly describe *classic* symptoms of depression (eg, fatigue, difficulty in concentrating, and loss of interest) than increases in negative affect, which include feelings of hostility and irritability. Therefore, the findings for our White non-Hispanic participants are in line with prior studies in this area that used mostly White samples, which are likely not generalizable to more diverse populations [57]. Overall, our study supports the idea that for this subsample of users, *passive* SMU is linked to indicators of depression.

However, considering that our participants were determined to be at least mildly depressed before the study, this result may be better understood as indicating that young people who are depressed are more likely to engage in *passive* SMU when experiencing lower levels of positive affect. Entertainment has been identified as the highest-rated motivation for SMU [58,59]. It seems that White youths with depression are likely to turn toward social media for entertainment and distraction when experiencing low levels of positive affect. Being actively engaged with others may not be congruent with such an affective state. Although this interpretation is consistent with our findings, our methods did not test the contributory relationship of this association. Additional research is necessary to fully reveal the complex interplay between SMU and momentary affect.

For Black non-Hispanic participants in our study, interacting directly with other people while using social media (*active* SMU) was associated with higher levels of negative affect. This finding is somewhat counter to other research showing that the active use of media is protective against symptoms of depression [26]. Although feelings of hostility and irritability are not necessarily indicators of depression, youths with depression have been shown to have higher levels of hostility both on the web and offline [60]. In our study, *active* SMU may have elicited an increase in negative affect among Black non-Hispanic youths who are depressed rather than providing a type of social support that could reduce negative symptoms.

Such a response by Black non-Hispanic adolescents may be explained by the dual nature of SMU for minority youths. The risk of experiencing racism-based cyberaggression, blatant acts of racism, microaggressions, and other forms of systemic oppression on social media, which can be further amplified by platform-based algorithms, may lead to different SMU experiences for minority-identifying individuals than for their White peers [61,62]. Lower levels of minority representation and fewer minority voices on social media, in general, may contribute to a less welcoming social media environment, leading to more negative affective experiences.

Even in web-based community building and activism, the positive side of the dual nature of SMU for minorities may hold experiences that could be more upsetting for Black non-Hispanic youths as they can include being exposed to, sharing experiences about, and building solidarity and organizing against racism and inequality. Previous research has demonstrated that Black and Hispanic participants report higher rates of *active* SMU in the form of political activism and that Black participants are more likely to engage actively with news stories posted on social media [63,64]. Considering the potential for exposure to race-related or underrepresentative news and political content on social media, it is reasonable to expect that activism and responses related to this material might contribute to greater negative affect. Although this interpretation may explain our findings, our study did not test it directly. Additional research is necessary to determine what aspects of *active* SMU might differ by race/ethnicity and translate into more negative affect for Black non-Hispanic youths.

Again, it is important to remember that our study did not test the direction of effect. Black non-Hispanic youths may turn to *active* SMU when they are experiencing higher levels of negative affect or *active* SMU may increase their negative affect or both. Given that Black non-Hispanic youths may have web-based social networks with a wider variety of relationship types (strong and weak) [45] than their White non-Hispanic peers, they may be more likely to have web-based social resources to cope with high levels of negative affect. Future research should examine how youths of different ethnicities differ in their motivations for and expectations of SMU to help understand whether and why minority youths are at a higher risk for poorer mental health outcomes.

### Research, Clinical, and Industry Implications

On the basis of these findings, researchers should investigate the possibility that SMU is not experienced uniformly. One’s racial/ethnic identity may be an important mediator of affective experiences during SMU. Although depression and anxiety have often been a major focus of research on the effects of SMU, there may be value in studying affect in greater detail, given the relationship between lower positive affect and depression and between higher negative affect driven by irritability/hostility and activist solidarity.

From a clinical standpoint, as associations between SMU and behavioral health conditions become more clear, clinicians will need to become comfortable with assessing and providing counseling on *problematic interactive media use* behaviors as they do with other well-established health risk behaviors. It may be clinically useful to consider how affective states may drive school or homework avoidance and/or excessive interactive media use. Conversely, we need to better understand individuals’ intentions and the context of SMU, as this may be a modifiable factor that can influence affective outcomes. Our society has grown increasingly dependent on the use of digital communication for critical life activities, from education to employment and communication to interpersonal connections and relationships. If differences exist in the overall experience and impact of SMU among different racial/ethnic groups, it will be important to understand these differences and translate them into effective screening and equitable treatment of patients.

If it is found that the design of social media platforms may contribute to inequitable SMU experiences or unhealthy behavioral outcomes, technology companies will have the opportunity to respond with design changes that promote greater equity and more positive mental health outcomes. In 2019, the social media platform Instagram started testing the potential value of hiding *like* counts to reduce user exposure to social evaluations that might threaten users’ self-esteem. Instagram’s chief executive officer Adam Mosseri promised, “We will make decisions that hurt the business if they help people’s well-being and health.” [65] In May 2021, after 2 years of testing, Instagram announced that although removing *likes* was beneficial for some users, it annoyed others, and it did not *meaningfully depressurize* their platform [66]. As a result, Instagram and parent company Facebook decided to offer users the option of turning off *likes* but leaving them viewable to others by default. Similar observations of different affective experiences of SMU by various racial/ethnic groups and considerations of design changes could improve digital wellness.

### Limitations

The study’s limitations include the relatively small sample size, which reduces the generalizability of the results. However, the sample included a diverse group of participants drawn from a population of patients with evidence of at least mild depression, which is an at-risk group of great clinical interest. In addition, our analyses were performed at the moment level, thereby increasing the statistical power.

Although the overall EMA survey response rate (approximately 50%) was similar to that of other studies using this methodology with adolescents, it does represent a potential selection bias. Participants with higher response rates could provide qualitatively different responses than those with lower response rates. For instance, participants with higher levels of social anxiety exhibited higher EMA response rates. In addition, adolescents may have avoided answering surveys during SMU, especially when they were actively engaged in it. However, EMA measures essentially eliminate recall bias, which likely greatly enhances the reliability of patient reports.

Another limitation of this study was the inability to prove that SMU contributes to mental health problems. Given the dynamic nature of social media, our results may be sensitive to the specific period during which the study was performed. Study participants’ SMU characteristics and affective experiences may be highly dependent on news stories that happened to be trending at a given point, creating the potential for chronology bias.

### Future Research

Moving forward, it will be important to measure momentary affective responses to SMU in more detail, with larger populations, with different social media designs, and over longer periods to achieve greater granularity and demonstrate the stability of these findings. Given the significant differences in affective experiences among racial/ethnic groups, future research should broaden to include gender, culture, and other experience-influencing user characteristics.

### Conclusions

At a time marked by increasing trends in mental health problems and unprecedented levels of interactive media use among children and adolescents, there is a real urgency to achieve a better understanding of the relationships between different media use behaviors and mental health problems. Identifying helpful and harmful digital design features and risk and protective factors in users will be of paramount importance, as will be the continued study of the role of racial/ethnic identities and other individual factors as potential moderators of media use experiences. As the nature of our social interactions continues to evolve in an increasingly digital landscape, so too must our understanding of the potential influence of these behaviors on the health and well-being of the children, adolescents, and young adults we serve.

## Abbreviations

EMA: ecological momentary assessment
PHQ-9: Patient Health Questionnaire-9
SMU: social media use
